# Incidence of pelvic inflammatory disease in a large cohort of women tested for *Chlamydia trachomatis*: a historical follow-up study

**DOI:** 10.1186/1471-2334-9-130

**Published:** 2009-08-14

**Authors:** Inger J Bakken, Sara Ghaderi

**Affiliations:** 1 Department of Preventive Health Care, SINTEF Technology and Society, Trondheim, Norway; 2 Department of Mathematical Sciences, Faculty of Information Technology, Mathematics and Electrical Engineering, Norwegian University of Science and Technology (NTNU), Trondheim, Norway; 3 Regional Centre for Child and Adolescent Mental Health, Department of Neuroscience, Norwegian University of Science and Technology (NTNU), Trondheim, Norway

## Abstract

**Background:**

*Chlamydia trachomatis *is a highly prevalent sexually transmitted disease. Testing rates among young Norwegian women are high. Young women diagnosed with *C. trachomatis *are often worried about future complications.

**Methods:**

Our cohort consisted of 24,947 women born 1970–1984 who were tested for *C. trachomatis *infection during 1990–2005. We linked *C. trachomatis *laboratory data to data on hospitalizations for pelvic inflammatory disease during 1990–2005. Cox regression analysis with time-dependent covariates adjusted for age at first test was used to assess the association between *C. trachomatis *history and pelvic inflammatory disease.

**Results:**

Follow-up until the end of 2005 included 201,387 woman-years. The incidence rate of hospitalization for pelvic inflammatory disease was higher among women with prior *C. trachomatis *infection than among women with negative tests only (48 events during 32,057 person-years and 143 events during 169,192 person-years, corresponding to 0.15 and 0.08 per 100 person-years, respectively). The corresponding hazard ratio adjusted for age at first test was 1.69 (95% CI, 1.21–2.36).

**Conclusion:**

Our data show a link between a diagnosis of *C. trachomatis *infection and subsequent pelvic inflammatory disease. However, pelvic inflammatory disease was a rare event irrespective of *C. trachomatis *status. These, together with other recent findings, can be used to reassure women worried about their future reproductive health following a diagnosis of *C. trachomatis*.

## Background

*Chlamydia trachomatis *is a highly prevalent sexually transmitted disease [1-3]. Although there is no systematic screening program in Norway, testing rates among young women are high, as are the positivity rates [4].

By using data from a laboratory that covers all chlamydial diagnostics in a defined geographical area (a Norwegian county), we have previously shown that at the age of 25, one in six women had been diagnosed with *C. trachomatis *at least once [4]. Chlamydial infections have potential long-term consequences such as pelvic inflammatory disease, tubal factor infertility, and ectopic pregnancy [5].

Women with positive test results are often anxious about their future fertility [6,7]. A recent review has however shown that the previously assumed high complication rates of *C. trachomatis *are now being increasingly questioned [8].

We have previously used a registry-linkage approach and applied a retrospective cohort design to investigate ectopic pregnancy rates and birth rates by test result among women tested for *C. trachomatis *in a routine clinical setting [9]. In the present study we have applied a similar design to assess the rate of pelvic inflammatory disease following a diagnosis of *C. trachomatis*.

## Methods

The study was carried out in Sør-Trøndelag County, central Norway. Of the county's 270,000 inhabitants approximately 150,000 are living in the major city, Trondheim.

### Data Sources

In Norway, all citizens are given a unique 11-digit social security number at birth or at immigration. In the current study, we used two databases, both containing the personal identifier and data on residency.

In Sør-Trøndelag County, a single laboratory is responsible for all *C. trachomatis *diagnostics [4]. The *C. trachomatis *database contains information on all tests (date, diagnostic method, and test outcome) in the county from November 1990 to December 2005 with person as the unit of analysis.

Information on all hospitalizations with a diagnosis of pelvic inflammatory disease was obtained from discharge registries 1990–2005 of the two hospitals in the county (Orkdal Hospital and Trondheim University Hospital). Patients with pelvic inflammatory disease were identified through computerized hospital in- and outpatient registries by using the International Classification of Diseases (ICD) 9th Revision code 614 during 1990–98 and the ICD 10th Revision codes N70 over the years 1999–2005.

### Laboratory Methods

Several methods were used for *C. trachomatis *detection throughout the study period[4] Briefly, the IDEIA™ Chlamydia Test, Celltech Diagnostics/Novo BioLabs/DAKO was replaced by PACE 2™, GenProbe in 1992, whereas Amplicor from Roche Molecular Systems became the routine detection method in 1999.

### Study Population

**Figure 1:**
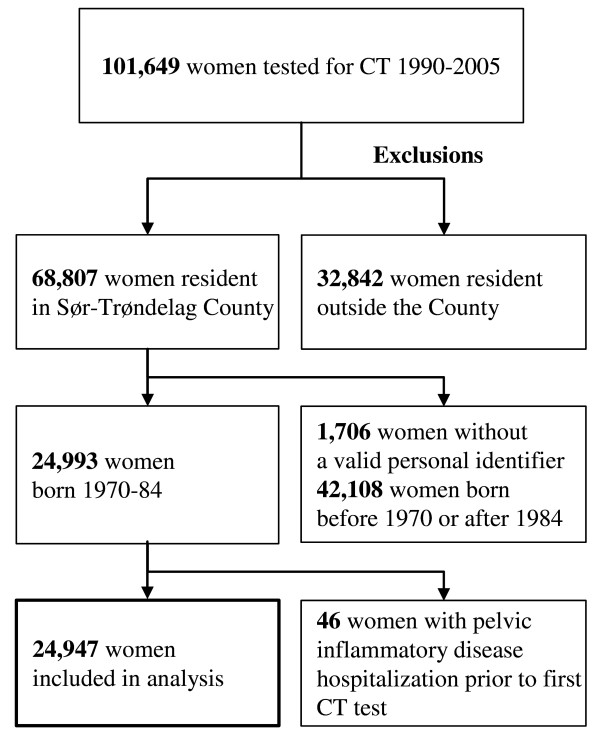
**Inclusions and exclusions of women to the study**.

During November 1, 1990, to December 31, 2005, 101,649 women (all ages included) were tested for *C. trachomatis*, among whom 68,807 resided in Sør-Trøndelag County (Figure 1). We excluded 1,706 women without a valid personal identifier. In order to obtain a study population with a nearly complete testing history, we limited the population to women who were 20 years old or younger when computerized registration of test results started in 1990. Thus, 42,108 women born before 1970 or after 1984 were excluded from the study population (Figure 1). We finally excluded 46 women who were registered with a hospitalization for pelvic inflammatory disease prior to their first registered *C. trachomatis *test. Thus, our study population consisted of 24,947 women who had not experienced a hospitalization for pelvic inflammatory disease prior to their first *C. trachomatis *test.

### Statistical Analysis

Confidence intervals (95%) for proportions were calculated using the Wilson method [10]. All other analyses were carried out using SPSS for Windows, version 15.0 (SPSS, Chicago, IL).

For estimation of hazard ratio for pelvic inflammatory disease by *C. trachomatis *status we used Cox regression analysis with months from the first *C. trachomatis *test as the time variable. *C. trachomatis *status was entered as a time-dependent covariate. *C. trachomatis *status changed at the date of a positive test. Analyses were adjusted for age at first test. Women were followed up until the first date of hospitalization with a diagnosis of pelvic inflammatory disease or censored on December 31, 2005.

### Ethics

The study adheres to the Helsinki Declaration and was approved by the Regional Committee for Research Ethics, Central Norway, and the Norwegian Data Inspectorate. Authorization for the use of laboratory data and data retrieved from medical records was obtained from the Norwegian Directorate of Health and Social Affairs.

## Results

The total follow-up time from time of first *C. trachomatis *infection until first hospitalization with a diagnosis of pelvic inflammatory disease or censoring was 201,249 person-years. The average duration of the follow-up period was 8.0 person-years (SD 4.6 person-years).

### Testing Pattern

A total of 93,089 tests were registered among the 24,947 women in our study cohort during the follow-up period. The average age at first test was 21.0 years (SD 3.7). A single test was registered for 6,717 women (27%). Four-thousand-seven-hundred-and-fifty women (19%) were registered with two tests, 3,500 (14%) women had three tests, 2,559 (10%) had four tests, and 7,421 women had five or more tests. There was a large variation in the time intervals between tests. We calculated the time interval between the first two tests and the second and the third test. For 5,408 women, both the time interval between the first two tests and the time interval between the second and the third test were 18 months or shorter, indicating that 22% of the total study population were screened on a fairly regular basis.

### Positive tests

Overall, the proportion of positive tests was 6.0% (5,577/93,089). The proportion of positive tests was highest early in the study period and the prevalence at first test was slightly higher than the total proportion of positive tests (Table 1).

**Table 1:** Total number of tests, positivity rates, number of first tests, and prevalence at first test among the women in the cohort

	1990–93	1994–96	1997–99	2000–03	2004–05
*All tests*					
Total number of tests	13,134	16,741	18,991	30,020	14,203
Proportion of positive tests	7.3%	6.2%	5.0%	6.2%	5.6%
*Restricted to first test for each woman*					
Number of first tests	6,761	4,588	4,480	6,049	3,069
Prevalence at first test	8.0%	7.6%	5.9%	8.2%	7.6%

During the observation period, 18% of the women in the cohort were infected with *C. trachomatis *at least once (4,413/24,947). A single infection was registered for 3,513 women (14%), 701 (3%) were registered with 2 infections, and 199 (1%) with 3 or more infections.

### Hospitalizations for PID

At least one hospitalization for pelvic inflammatory disease was registered for 191 out of the 24,947 women in the cohort (0.77%).

Forty-eight out of the 4,413 women with prior *C. trachomatis *infection experienced hospitalization with a diagnosis of pelvic inflammatory disease (1.09%; 95% CI, 0.82–1.44). The corresponding number among the 20,534 women without prior infection was 143 (0.70%; 95% CI, 0.59–0.82).

As shown in Table 2, the incidence rate of hospitalization with a diagnosis of pelvic inflammatory disease was higher for women with one or more prior infections compared to women with negative tests only (hazard ratio, 1.69; 95% CI 1.21–2.37).

**Table 2:** Cox regression analysis of hospitalizations with pelvic inflammatory disease by previous *C. trachomatis *infection

Prior infections	No. of women*	Observation years	No. of events	Incidence rate per 100 person-years (95% CI)	Adjusted† hazard ratio (95% CI)
None	20,534	169,192	143	0.08 (0.07–0.10)	Reference
One or more	4,413	32,057	48	0.15 (0.11–0.20)	1.69 (1.21–2.37)

* Number of women at end of follow-up period

† Adjusted for age at first test

## Discussion

This registry-based cohort study shows that women diagnosed with *C. trachomatis *infection had a slightly higher risk for hospitalization with a diagnosis of pelvic inflammatory disease compared to women with only negative tests.

The present study is unique in that we had access to data on all *C. trachomatis *tests over a long time period (1990–2005) within a well-defined geographic region (county) for a young cohort with a high testing frequency [4], at least compared to previous registry studies [11,12]. By comparison with data from Statistics Norway, we estimate that our population represents approximately 80% of all women born 1970–84 in the study area (data not shown). We identified all cases of pelvic inflammatory disease through hospital in-and outpatient registries.

The study's main limitation is that we had no access to data from primary care. Mildly asymptomatic cases treated outside hospital settings or asymptomatic cases are missed. Although such cases also might have potential for long-term adverse reproductive health effects, hospitalizations are likely to reflect the most severe cases of pelvic inflammatory disease. Another limitation of our study is that the study population consists of women tested for *C. trachomatis *in a routine clinical setting. Because the women were not systematically screened, the number of tests for each woman varied, as did the interval between tests. Furthermore, over the study period, there was a change in test technology towards more sensitive tests. Finally, we did not have access to other data of potential interest, such as sexual activity and contraception use.

We found a higher incidence rate of pelvic inflammatory disease among women diagnosed with *C. trachomatis *infection than among women with negative tests only. This result is in concordance with results from a Swedish registry-linkage study [12]. The risk estimate was however slightly lower in that study compared to ours, possibly because we had access to more recent and complete data on both *C. trachomatis *testing and pelvic inflammatory disease hospitalizations (1990–2005 for both exposure and outcome). In the Swedish study, the *C. trachomatis *database covered the time period 1985–1996, while outcomes were assessed until 1999. Missing data on testing history and a large proportion of tests analyzed with less sensitive technology will tend to bias the results towards the null because of misclassification of infection status.

Although the present study showed higher incidence rates of pelvic inflammatory disease among women with prior positive tests than among women with negative tests only, the rates were low in either group. It has been suggested that the relatively low incidence of lower genital tract complications among women diagnosed with *C. trachomatis *infection indicates that the benefits of chlamydia screening programs might have been overestimated [12]. However, the present and previous registry studies include women tested for *C. trachomatis *in routine clinical settings [9,11-13], and it is most unlikely that a large proportion of women with a diagnosis were left untreated. Thus, the high prevalence rate of infection together with the low incidence rates of complications among women with positive tests might rather indicate that screening for chlamydial infection benefits female reproductive health. Such findings do however show that women with positive test results, often anxious about their future fertility [6,7], can be reassured that the risk of long term reproductive health consequences is low after a treated infection.

## Conclusion

The present study shows a clear link between diagnosed *C. trachomatis *infection and subsequent pelvic inflammatory disease. However, hospitalization for pelvic inflammatory disease was a rare event also among women with prior infections. This, held together with previous findings of low ectopic pregnancy rates also among women with prior infection, might indicate that the large-scale opportunistic screening for *C. trachomatis *over the last decades has enhanced the reproductive health of Norwegian women.

## Competing interests

IJB has received an honorarium for a lecture from Puls Norway AS, a company that distributes *C. trachomatis *diagnostic material.

## Authors' contributions

IJB conceived of the study, linked the data, was the main contributor to the data analysis, and drafted the manuscript. SG contributed to data analysis and helped draft the manuscript. Both authors read and approved the final manuscript.

## Pre-publication history

The pre-publication history for this paper can be accessed here:

http://www.biomedcentral.com/1471-2334/9/130/prepub

## References

[B1] Fenton KA Lowndes CM Recent trends in the epidemiology of sexually transmitted infections in the European Union Sex Transm Infect 2004 80 255 263 10.1136/sti.2004.009415 15295121 PMC1744866

[B2] Gaydos CA Howell MR Pare B Clark KL Ellis DA Hendrix RM Chlamydia trachomatis infections in female military recruits N Engl J Med 1998 339 739 744 10.1056/NEJM199809103391105 9731090

[B3] La Montagne DS Patrick LE Fine DN Marrazzo JM Re-evaluating selective screening criteria for Chlamydial infection among women in the U S Pacific Northwest Sex Transm Dis 2004 31 283 289 10.1097/01.OLQ.0000124613.85111.6B 15107630

[B4] Bakken IJ Nordbø SA Skjeldestad FE *C. trachomatis *testing patterns and prevalence of genital chlamydial infection among young men and women in central Norway 1990–2003: A population-based registry study Sex Transm Dis 2006 33 26 30 10.1097/01.olq.0000187929.36118.d2 16385219

[B5] Farquhar CM Ectopic pregnancy Lancet 2005 366 583 591 10.1016/S0140-6736(05)67103-6 16099295

[B6] Duncan B Hart G Scoular A Bigrigg A Qualitative analysis of psychosocial impact of diagnosis of Chlamydia trachomatis: implications for screening BMJ 2001 322 195 199 10.1136/bmj.322.7280.195 11159612 PMC26583

[B7] Kangas I Andersen B Olesen F Moller JK Ostergaard L Psychosocial impact of Chlamydia trachomatis testing in general practice Br J Gen Pract 2006 56 587 593 16882376 PMC1874522

[B8] Wallace LA Scoular A Hart G Reid M Wilson P Goldberg DJ What is the excess risk of infertility in women after genital chlamydia infection? A systematic review of the evidence Sex Transm Infect 2008 84 171 175 10.1136/sti.2007.026047 18055580

[B9] Bakken IJ Skjeldestad FE Lydersen S Nordbø SA Births and ectopic pregnancies in a large cohort of women tested for Chlamydia trachomatis Sex Transm Dis 2007 34 739 43 17479068 10.1097/01.olq.0000261326.65503.f6

[B10] Altman DG Machin D Bryant TN Gardner MJ Statistics with confidence 2000 2 UK: BMJ Books, London

[B11] Andersen B Ostergaard L Puho E Skriver MV Schonheyder HC Ectopic pregnancies and reproductive capacity after Chlamydia trachomatis positive and negative test results: a historical follow-up study Sex Transm Dis 2005 32 377 381 10.1097/01.olq.0000154512.86651.07 15912085

[B12] Low N Egger M Sterne JA Harbord RM Ibrahim F Lindblom B Incidence of severe reproductive tract complications associated with diagnosed genital chlamydial infection: the Uppsala Women's Cohort Study Sex Transm Infect 2006 82 212 218 10.1136/sti.2005.017186 16731670 PMC2576723

[B13] Bakken IJ Skjeldestad FE Nordbo SA Chlamydia trachomatis infections increase the risk for ectopic pregnancy: a population-based, nested case-control study Sex Transm Dis 2007 34 166 169 10.1097/01.olq.0000230428.06837.f7 16837829

